# How does shading mitigates the water deficit in young *Hymenaea courbaril* L. plants?

**DOI:** 10.3389/fpls.2023.1235234

**Published:** 2023-09-19

**Authors:** Lucas C. Reis, Silvana P. Q. Scalon, Andressa C. Foresti, Daiane M. Dresch, Cleberton C. Santos, Vânia T. Lima

**Affiliations:** ^1^ Plant Metabolism and Nutrition Laboratory, Faculty of Agricultural Sciences, Federal University of Grande Dourados, Dourados, State Mato Grosso do Sul, Brazil; ^2^ Departament of Botany, Biosciences Institute, São Paulo State University Júlio de Mesquita Filho, Rio Claro, São Paulo, Brazil

**Keywords:** photosynthesis, chlorophyll a fluorescence, antioxidant enzymes, proline, stresses abiotic

## Abstract

Information on tolerance to isolated or combined abiotic stresses is still scarce for tree species, although such stresses are normal in nature. The interactive effect of light availability and water stress has been reported for some native tree species in Brazil but has not been widely investigated. To test the hypothesis that shading can mitigate the stressful effect of water deficit on the photosynthetic and antioxidant metabolism and on the growth of young *Hymenaea courbaril* L. plants, we evaluated the following two water regimes: a) continuous irrigation – control (I) - 75% field capacity. and b) water deficit (S), characterized by irrigation suspension associated the two following periods of evaluation: P0 - when the photosynthetic rate of plants subjected to irrigation suspension reached values ​​close to zero, with the seedlings being re-irrigated at that moment, and REC - when the photosynthetic rate of the re-irrigated plants of each shading levels reached values ​​similar to those of plants in the control treatment, totaling four treatments: IP0, SP0, IREC, and SREC. The plants of these four treatments were cultivated under the four following shading levels: 0, 30, 50, and 70%, constituting 16 treatments. Intermediate shading of 30 and 50% mitigates the water deficit and accelerates the recovery of *H. courbaril*. Water deficit associated with cultivation without shading (0%) should not be adopted in the cultivation or transplantation of *H. courbaril*. After the resumption of irrigation in the REC, the other characteristics presented a recovery under all cultivation conditions. Key message: Intermediate shading of 30 and 50% mitigates the water deficit and accelerates the recovery of *H. courbaril*.

## Introduction

Shading, as well as water availability, are directly related to plant growth in a species-dependent relationship. Information on tolerance to isolated or combined abiotic stresses is still scarce for native species, although such stresses are normal in nature. The interactive effect of light, shading, and water stress has been reported for some native tree species in Brazil but has not been widely investigated, requiring further studies (Araújo et al., 2020; Bartieres et al., 2020; Cremon et al., 2020; Barbosa et al., 2021; Rosa et al., 2021).

The low water availability in the soil affects several metabolic processes, leading to smaller leaf areas, stomatal closure, lower stomatal conductance, lower rates of photosynthesis and transpiration, reduced growth, and lower yields. These conditions affect the enzymatic activity of carboxylase, ATPase, and antioxidant enzymes (Bartieres et al., 2020; Rosa et al., 2021). Species behave differently after the suspension of water stress; some do not survive, and others take time to recover, and other recover normally. These responses are attributed to physiological and metabolic plasticity, which ensures adjustments and tolerance to stressful environments (Araújo et al., 2020; Bartieres et al., 2020; Cremon et al., 2020; Barbosa et al., 2021; Rosa et al., 2021).

The physiological mechanisms responsible for the adaptations of native plants to shade or sun have not yet been widely evaluated and clarified. Thus, one same species is frequently classified in different successional groups. Ortiz et al. (2021) observed in their review that the functional characteristics of leaves vary between pioneer and late-successional species and that, among other traits, photosynthetic capacity and tolerance to photoinhibition are of great scientific interest (Yang et al., 2020). They also observed that pioneer species present a high capacity to dissipate excess light energy and to protect themselves by increasing carotenoids, modulating photosystem II (PSII), and by presenting a high content of the photosynthetic enzyme Rubisco. However, late-successional species are prone to chlorophyll degradation, exhibiting photoinhibition and presenting an irreversible decline in the maximum photochemical efficiency of PSII (F_v_/F_m_) when absorbing more light than necessary for carbon assimilation. In addition, late-successional species present lower Rubisco contents, which can eventually compromise plant survival (Yang et al., 2020; Ortiz et al., 2021).

Seedling growth in nurseries is directly influenced by factors such as water, temperature, relative humidity, and luminosity. Knowledge on the behavior of plants in the face of environmental factors is important, as the need to increase and diversify seedling production both for conventional agriculture and for the recovery of areas of natural vegetation degraded by human activity is growing throughout the world (Sousa et al., 2020).

Among the potential species for planting, there is the *Hymenaea courbaril* L., which is a species widely distributed in the Neotropical region and tolerant to different environmental conditions (Nascimento et al., 2015). *H. courbaril* is widely exploited due to its commercial and medicinal value and the properties of its wood, sap, bark, fruit, and seeds, which are highly valued. Thus, for its characteristics, this species is a promising candidate for reforestation programs or regeneration of natural habitats (Nascimento et al., 2015; Sousa et al., 2020). Oliveira et al. (2011) observed in their review that *H. courbaril* is included among the 25 priority species for programs to recover areas of degraded forest.

To understand the ability of plants of *H. courbaril* to acclimatize to the water deficit and the effect of shading on the mitigation of stress becomes relevant to ensure the successful implementation of projects for the recovery of degraded areas, the enrichment of these areas or planting for sustainable exploitation. Thus, taking into account the hypothesis that shading can mitigate the stressful effect of water deficit on the photosynthetic and antioxidant metabolism and on the growth of plants of *Hymenaea courbaril* L., we evaluated the behavior of young plants under water deficit at different shading levels and their recovery potential after the resumption of irrigation.

## Materials and methods

### Study site and species

The experiment was carried out at the Faculty of Agricultural Sciences (22°11’43.7” S and 54°56’08.5” W, altitude of 452 m), Federal University of Grande Dourados (UFGD), municipality of Dourados, MS, Brazil.

Ripe fruits of *H. courbaril* were collected from matrices in the remaining areas of the Cerrado. Subsequently, we manually processed and sanitized the seeds in 2% sodium hypochlorite for 5 min. The seeds were scarified with emery to overcome tegumentary dormancy and immersed in water for 24 hours before soaking (Souza et al., 2015). Sowing was performed in polyethylene tubes with a volume of 290.0 cm^3^ filled with commercial substrate Tropstrato had the following attributes: pH CaCl_2_, 5.75; P, 65.70 mg dm^−3^; K, 1.60 cmol_c_ dm^−3^; Ca, 23.80 cmol_c_ dm^−3^; Mg, 12.40 cmol_c_ dm^−3^; Al, 0 cmol_c_ dm^−3^; H + Al, 4.20 cmol_c_ dm^−3^; the sum of bases, 39.80 cmol_c_ dm^−3^; cation exchange capacity, 42.10 cmol_c_ dm^−3^; and base saturation (V%), 64.80, and irrigation was performed daily according to Souza et al. (2000).

At 15 days after emergence, the seedlings were transplanted into 7.0 L plastic pots, previously filled with an Oxisol soil (United States and Soil Survey Staff, 2014) that corresponds to a Distroferric Red Latosol in the Brazilian Soil Classification System (EMBRAPA, 2018) and coarse sand (3:1, v/v). The substrate was maintained 70% of the water retention capacity (WRC) (Souza et al., 2000) until plant reached an average height of 24.0 cm for 30 days (acclimatization).

### Water regimes, shading levels, and design

The plants with 45 days old were separated into four groups based on the following water regimes: continuous irrigation (control) – I, in which 75% of the water-holding capacity was maintained in the substrate using the gravimetric method (Souza et al., 2000); and water deficit – S, characterized by irrigation suspension. The water regimes were associated with the following evaluation periods: P0 - when the photosynthetic rate of young plants subjected to irrigation suspension (S) reached values ​​close to zero, with the plants being re-irrigated at that moment; and REC - when the photosynthetic rate of the re-irrigated plants at each shading level reached values ​​similar to those of plants in the irrigated with the same level of shading. Thus, the following treatments were established: 1) irrigated at zero photosynthesis (IP0), 2) stressed at zero photosynthesis (SP0); 3) irrigated in recovery (IREC); and 4) stressed in recovery (SREC). Zero photosynthesis was determined when plants subjected to water deficit any shading level reached values close to zero.

The young plants of these four treatments were allocated in the following four shading levels: 0% (0 = full sun), 30% (30), 50% (50), and 70% (70), obtained with the use of black nylon screens (Sombrite^®^) in a greenhouse. All the plants were maintained under a plastic screen to avoid the precipitation that could occur during the period.

The treatments were arranged in a completely randomized design in a split-plot scheme, where water regimes associated with evaluation periods (IP0, SP0, IREC, and SREC) were allocated in the plots and the different shading levels (0, 30, 50, and 70%) were allocated in the subplots, with six replicates (*n* = 6), with two plants by pot, totaling 96 plants.

### Measurements

#### Gas exchanges and chlorophyll *a* fluorescence

Gas exchanges and fluorescence were determined between 8:00 and 11:00h in leaves of the third pair (from apex to base), and the evaluations were carried out in the morning, between 8 a.m. and 11 a.m. The light response curve was measured and the saturation point of the photosynthetic system was determined, establishing the value of the photosynthetic photon flux density (PPFD) of 1,300; 700; 500 and 400 μmol m^-2^ s^-1^ PPFD under 0, 30, 50, and 70% shading, respectively, and CO_2_ atmospheric ≥ 400 ppm as the ideal to perform the analyses.

The gas exchange were measured using a portable photosynthesis meter, LCIPro-SD ADC Bio Scientific Ltd., through which the photosynthetic rate (*A* - µmol m^-2^ s^-1^), stomatal conductance (*g*_s_ -mol m^-2^ s^-1^), internal CO_2_ concentration (*C*_i_ -µmol mol^-1^), Rubisco carboxylation rate (*A/C*_i_), and water use efficiency (*A/E*) were evaluated. Measurements were performed on fully expanded leaves of the third pair (from apex to base). The evaluations were carried out in the morning, between 8 a.m. and 11 a.m.

The potential quantum efficiency of photosystem II (F_v_/F_m_) was evaluated using a portable fluorometer, model OS-30p (Opti-Sciences Chlorophyll Fluorometer, Hudson, USA) under flash 1,500 from 1 s to 1,500 μmol m^−2^ s ^−1^, and from the measurements of initial fluorescence (F_0_) and maximum fluorescence (F_m_), absorbed energy conversion efficiency (F_v_/F_0_) and the basal quantum yield of non-photochemical processes in the PSII (F_0_/F_m_) were evaluated.

For fluorescence, the leaves were subjected to a period of 30 minutes of adaptation to the dark with the help of dark adapter clips, so that all reaction centers in this leaf region acquired the “open” condition that is, presenting a complete oxidation of the photosynthetic electron transport system.

#### Photosynthetic pigments

The contents of chlorophyll “a”, “b”, total (µg cm²) and carotenoids (µg cm²) were quantified by collecting a fully expanded leaf blade per plant. Samples were collected and kept in a dark and refrigerated environment. The entire extraction and quantification process was performed in the laboratory without direct exposure to light. 1 g was weighed, macerating with a pestle in a mortar, in 8 mL of 80% acetone, adding MgCl_2_ during the extraction. Subsequently, the solutions were taken for centrifugation using a microcentrifuge (MCD–200, H.T.) at a speed of 1.500 rpm for 10 minutes. Then, the absorbance reading was performed at the wavelengths of 470, 645 and 663 nm, using a spectrophotometer (SP-220, Biospectro). The chlorophyll and carotenoid contents respectively were found according to the proposals Arnon (1949) and Lichtenthaler and Buschman (2001), respectively.

#### Total protein, and antioxidant enzyme activity in leaves and roots

The total protein in leaves and roots was measured through quantitative determination, using the method by Bradford (1976). The fresh samples were used in the extract preparation. Absorbance readings were performed in triplicate at a wavelength of 595 nm using a spectrophotometer (Metash Visible Spectrophotometer- model V5000).

After harvesting the fresh leaves and roots of the treatments, the material was frozen in liquid nitrogen. One gram of each sample was weighed to be macerated in 6 mL of a solution containing 0.3 g polyvinylpyrrolidone (PVP) diluted in 100 mL of potassium phosphate buffer (0.2 M). Then, the samples were centrifuged at 4000 rpm for 20 minutes and the supernatant was used as an enzyme extract (Broetto, 2014). The activity of the antioxidant enzymes superoxide dismutase, and peroxidase, was measured in leaf and root tissues, following the methodology compiled by Broetto (2014).

#### Relative water content in leaves

The relative water was determined in four leaves of each treatment according to the following mathematical expression:


$$RWC=100\times \frac{\left(\text{fresh mass}-\text{dry mass}\right)}{\left(\text{saturated mass }-\text{ dry mass}\right)},$$

The leaves were collected between 7:00 and 10:00h and cut with cylinders of known area. After weighing the fresh mass, the samples were placed in petri dishes with distilled water for 24 h for saturation. After weighing the saturated dishes, they were dried to determine the dry mass.

#### Proline content

The leaf samples were dried in an oven with forced air circulation at 60 ± 5°C by 72 hours, and ground with sulfosalicylic acid according to the methodology of Colton-Gagnon et al. (2014). Absorbance at 520 nm (Spectrophotometer Metash V5000) was measured using toluene as a reference. Free proline content was calculated according to Bates et al. (1973).

#### Growth and seedling quality

Root length was measured using a ruler graduated in centimeters and the results were expressed in centimeters. Leaf area was measured using a Li-3100 Area Meter and the results were expressed in cm^2^. as a function of shoot height (SH), collar diameter (D), and total dry matter (TDM) which is given by the sum of shoot dry matter (SDM) and root dry matter (RDM). The seedlings were dried in a forced air circulation oven at 70°C ± 2°C for 72 hours until reaching constant mass and measured using an analytical scale (0.0001g).

Seedling quality was determined using the Dickson Quality Index (DQI) Quality was evaluated using the following equation Dickson et al. (1960) $\;DQI=\frac{TDM}{\left(\frac{SH}{D}\right)+\left(\frac{RDM}{SDM}\right)}$ .

### Data analysis

Data were submitted to analysis of variance (ANOVA), and when significant by the F test (p ≤ 0.05) the means for water regimes + periods of evaluation, shading levels, and the interaction of treatments, were compared by the Tukey test (*p* ≤ 0.05). The software SISVAR 5.6 was used for the analyses (Ferreira, 2011).

## Results

We observed that in P0, regardless of the shading level, the *A* was lower than the control treatment, sendo que as plantas que primeiro apresentaram F0 foram aquelas submetidas ao déficit hídrico sob pleno sol (10 dias de suspensão da irrigação). Under 50% shading level, *A* was 86.27% higher compared with plants under the 0% shading level.

**Figure 1:**
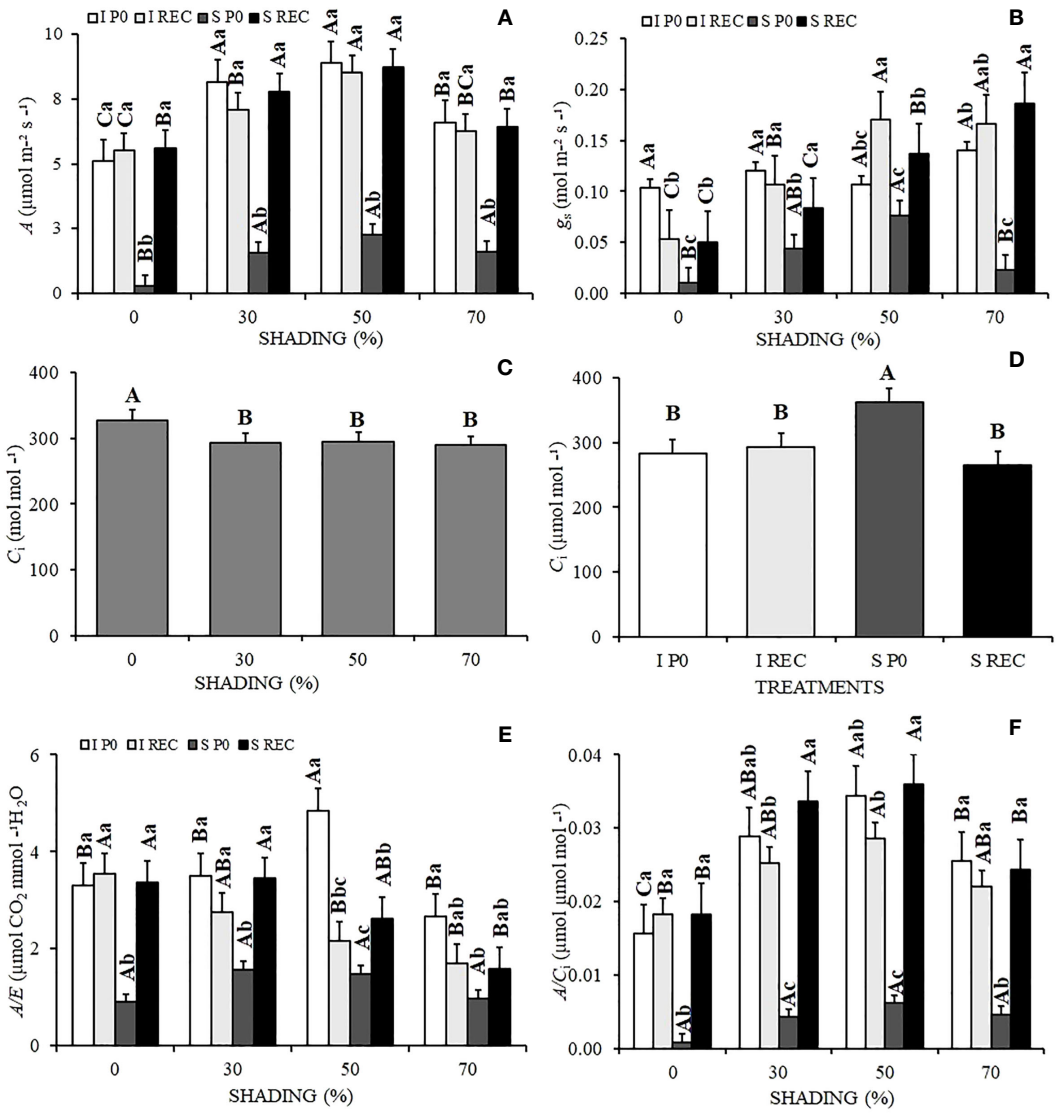
Mean values of the photosynthetic rate *A* **(A)**, stomatal conductance *Gs* **(B)**, internal CO_2_ concentration *C*_i_ **(C, D)**, instantaneous water use efficiency *A/E* **(E)**, and instantaneous efficiency of carboxylation of CO_2_ *A/C*_i_ **(F)** of *Hymenaea courbaril* L. seedlings under different water regimes + evaluation periods: irrigated at zero photosynthesis (IP0), stressed at zero photosynthesis (SP0); irrigated in recovery (IREC), stressed in recovery (SREC) under different shading levels: 0, 30, 50, and 70%. Capital letters compare the same water regime + evaluation period in the different shading levels. Lowercase letters compare the same shading level in the different water regimes + evaluation periods. Means followed by different letters indicate significant differences by the Tukey`s test (p<0.05) (*n*= 3).

The recovery of times of *A*, occurred differently, leading to different times being 8, 6, 3 and 5 days, for the respective shading 0, 30, 50, and 70%. In the SREC, overy stage (REC), after the establishment of irrigation, the plants previously stressed under 30 and 50% shading levels showed a higher *A* than that observed in plants under 0 and 70% shading levels (**Figure 1A**).

The stomatal conductance (*Gs*) was also significantly reduced at P0 in plants under water deficit for all shading levels, although remaining higher in seedlings grown under 50% shading, not differing statistically from plants under 30% shading. However, in REC, plants subjected to stress and 70% shading presented a significant increase in the rates of *Gs*, which were 26.79% higher than seedlings under 50% shading in the same water regime and period of evaluation (**Figure 1B**). Under the highest shading levels (50 and 70%), *Gs* was higher when compared to the lowest levels, except for P0.

There was no interaction between treatments for the internal carbon concentration (*C*_i_), which was higher in plants grown under 0% shading (**Figure 1C**) and under water deficit at P0 (SP0) (**Figure 1D**). The water use efficiency (*A/E*), similarly to *A* and *Gs*, reduced significantly in the plants under water deficit at P0. However, there was no significant difference between shading levels. In REC, plants previously cultivated under water deficit under the shading levels of 0 and 30% showed significantly higher recovery (**Figure 1E**).

The Rubisco carboxylation rate (*A/C*_i_) at P0 was also significantly reduced for SP0 plants, although there was also no variation between shadings levels. The shaded seedlings presented a higher *A/Ci*, although the SREC was higher under 30 and 50% shading (**Figure 1F**).

**Figure 2:**
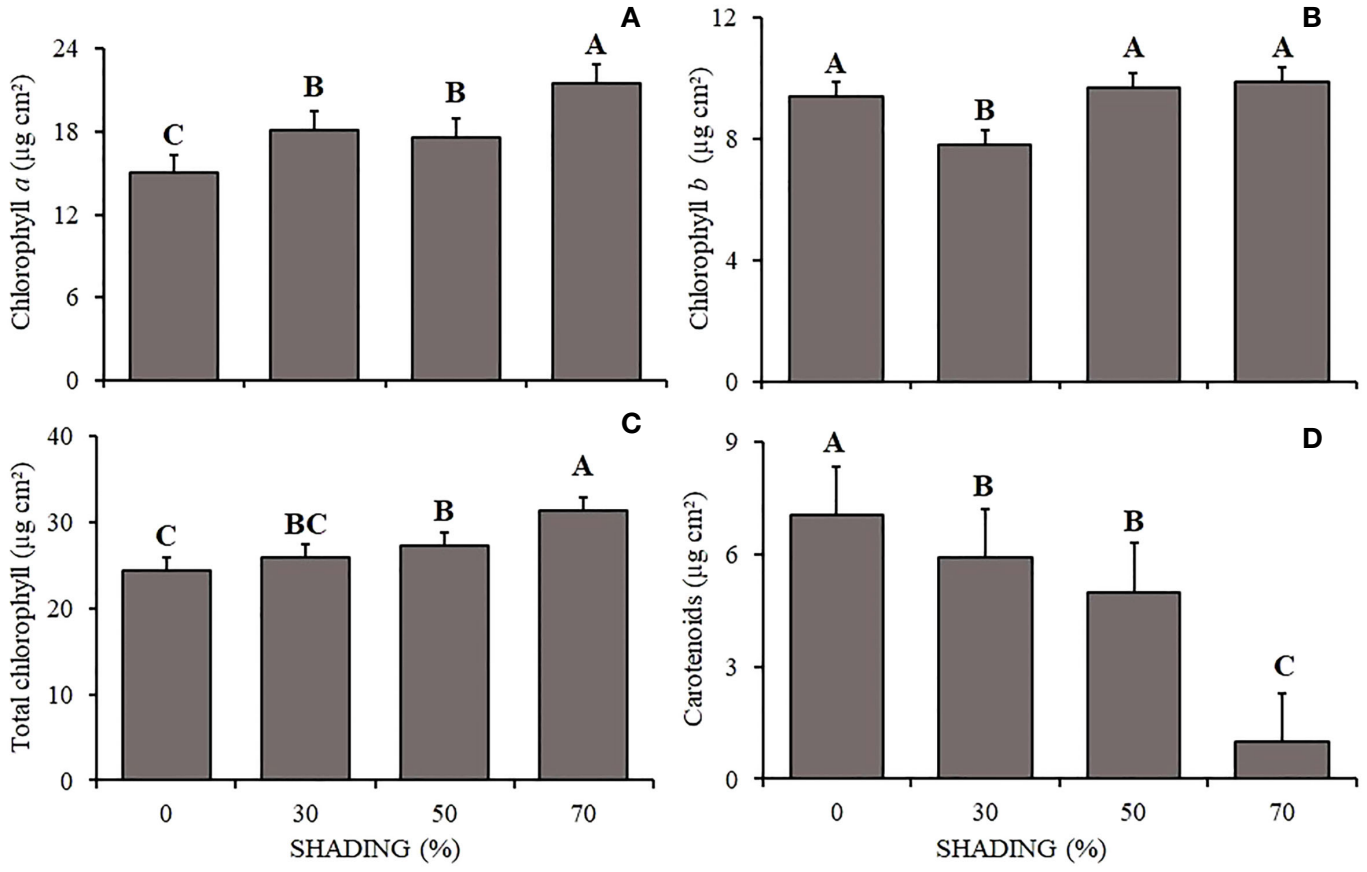
Mean values ​​of Chlorophyll a **(A)**, Chlorophyll *b* **(B)**, total Chlorophyll **(C)** and carotenoids **(D)** (µg cm²) of *Hymenaea courbaril* L. seedlings under different shading levels: 0, 30, 50, and 70%. Means followed by different letters indicate significant differences by the Tukey`s test (p<0.05) (*n*= 3).

The chlorophyll *a* content (**Figure 2A**) varied between shading levels, being higher in plants under 70% and lower under 0% shading. Chlorophyll *b* (**Figure 2B**) was lower in plants under 30% shading and did not vary in the other shading levels. Total chlorophyll (**Figure 2C**) followed the pattern of chlorophyll *a*, being higher in plants under 70% shading and lower under 0% shading. Carotenoids (**Figure 2D**) showed the opposite behavior, being higher in plants under 0% shading and lower under 70% shading.

**Figure 3:**
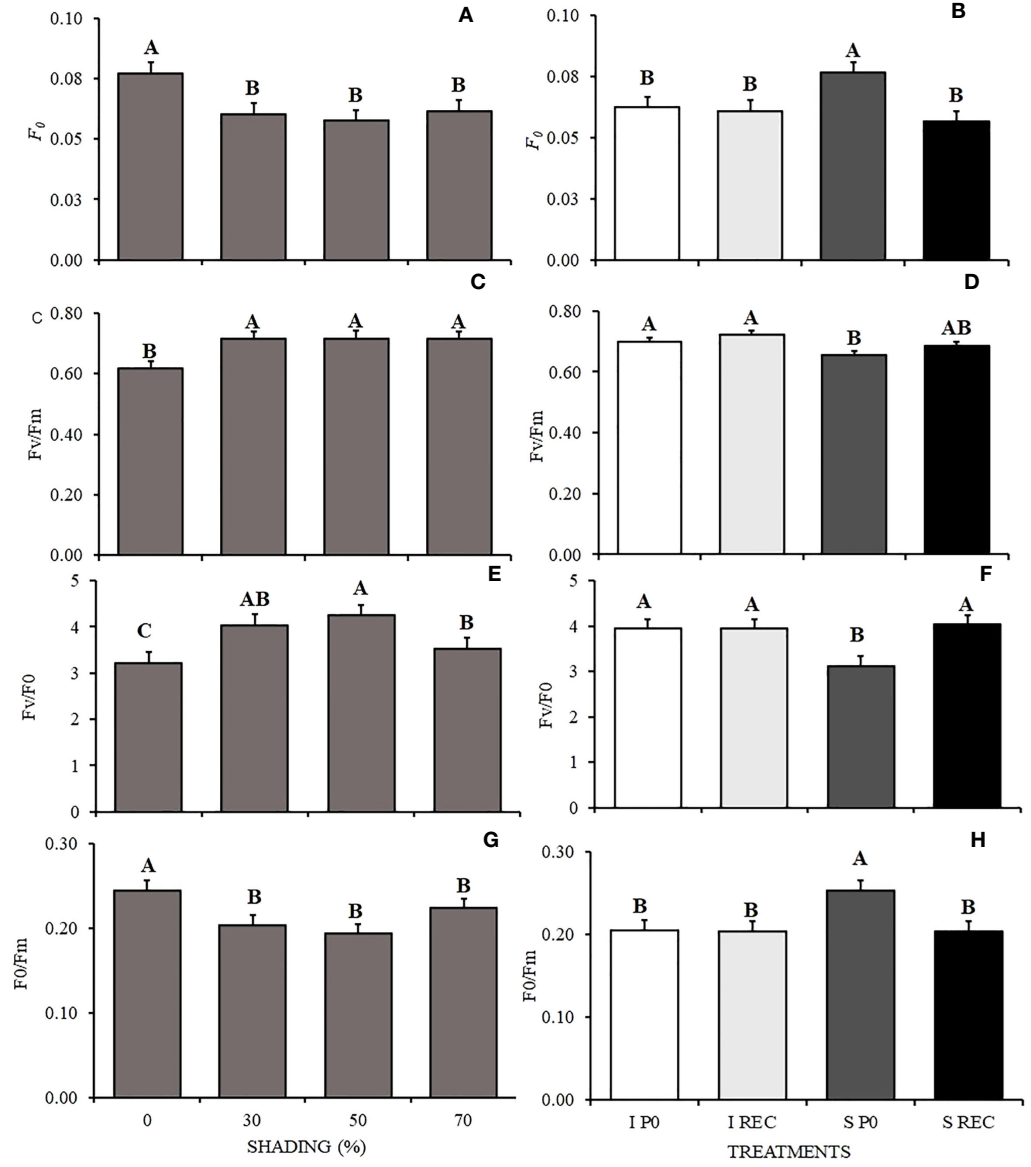
Mean values ​​of initial fluorescence - F_0_ **(A, B)**, quantum efficiency of photosystem II - F_v_/F_m_ **(C, D)**, efficiency of conversion of absorbed energy – Fv/F0 **(E, F)**, and ineffective photochemical processes of photosystem II - F0/Fm **(G, H)** in seedlings of *Hymenaea courbaril* L. under different water regimes + evaluation periods: irrigated at zero photosynthesis (IP0), stressed at zero photosynthesis (SP0); irrigated in recovery (IREC), stressed in recovery (SREC) under the following different shading levels: 0, 30, 50 and 70%. Means followed by different letters indicate significant differences by the Tukey`s test (p<0.05) (*n*= 3).

The plants under 0% shading showed higher initial fluorescence (F_0_) and in relation to the periods of evaluation, the highest value was observed for the SP0. In REC, regardless of the water regime, the values were lower (**Figures 3A, B**). Unlik P0, the quantum efficiency of photosystem II (F_v_/F_m_) showed lower values in plants under 0% shading and in SP0 (**Figures 3C, D**). The absorbed energy conversion efficiency (F_v_/F_0_) varied according to shading levels, being higher in plants under 30 and 50% shading and lower in plants under 0% shading. For the water regimes + evaluation periods, plants of the SP0 treatment showed lower values (**Figures 3E, F**).

However, for the basal quantum yield of the non-photochemical processes in the PSII (F_0_/F_m_) the opposite occurred, as plants under 0 and 70% shading presented higher values than the others, as well as the plants of the SP0 treatment (**Figures 3G, H**).

**Figure 4:**
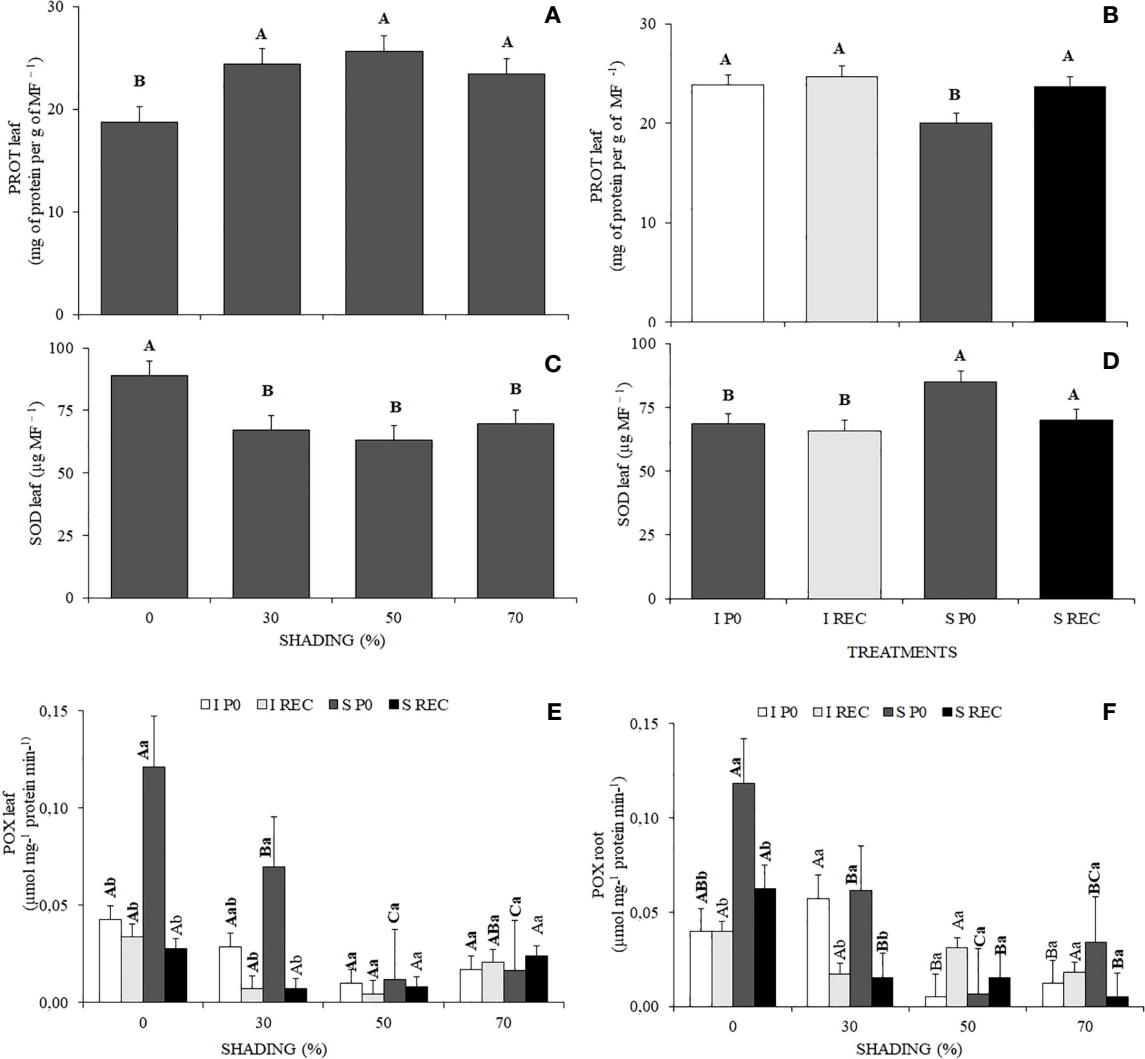
Mean values ​​of protein in the leaf **(A, B)**, activity of the enzymes in leaf superoxide dismutase (SOD) **(C, D)**, peroxidase (POX) **(E)** and root **(F)** of *Hymenaea courbaril* L. seedlings under different water regimes+ evaluation periods: irrigated at zero photosynthesis (IP0), stressed at zero photosynthesis (SP0); irrigated in recovery (IREC), stressed in recovery (SREC) under the following different shading levels: 0, 30, 50, and 70%. Capital letters compare the same water regime + evaluation periods in the different shading levels. Lowercase letters compare the same shading level in the different water regimes evaluation periods. Means followed by different letters indicate significant differences by the Tukey`s test (p<0.05) (*n*= 3).

Plants under 0% shading presented lower values of protein in leaves compared with the other shading levels. For the water regimes + evaluation periods, plants of the SP0 treatment presented lower values of protein in the leaves (**Figures 4A, B**).

The enzyme activity of superoxide dismutase (SOD) in leaves was higher in plants under 0% shading, as well as in plants of the SP0 treatment (**Figures 4C, D**).

The enzyme activity of peroxidase (POX) in leaves was higher in plants under 0% shading in the SP0 treatment, being like that of plants under 30% shading. However, in both shading conditions after the resumption of irrigation in the SREC,

POX levels reduced significantly, reaching values equal to the plants of the control treatment (**Figure 4E**). In Root, POX activity was also higher in plants under 0% shading in the SP0 treatment, followed by plants under 30% shading. However, in REC, the activity of this enzyme reduced significantly, although remaining higher in plants under 0% shading (**Figure 4F**).

**Figure 5:**
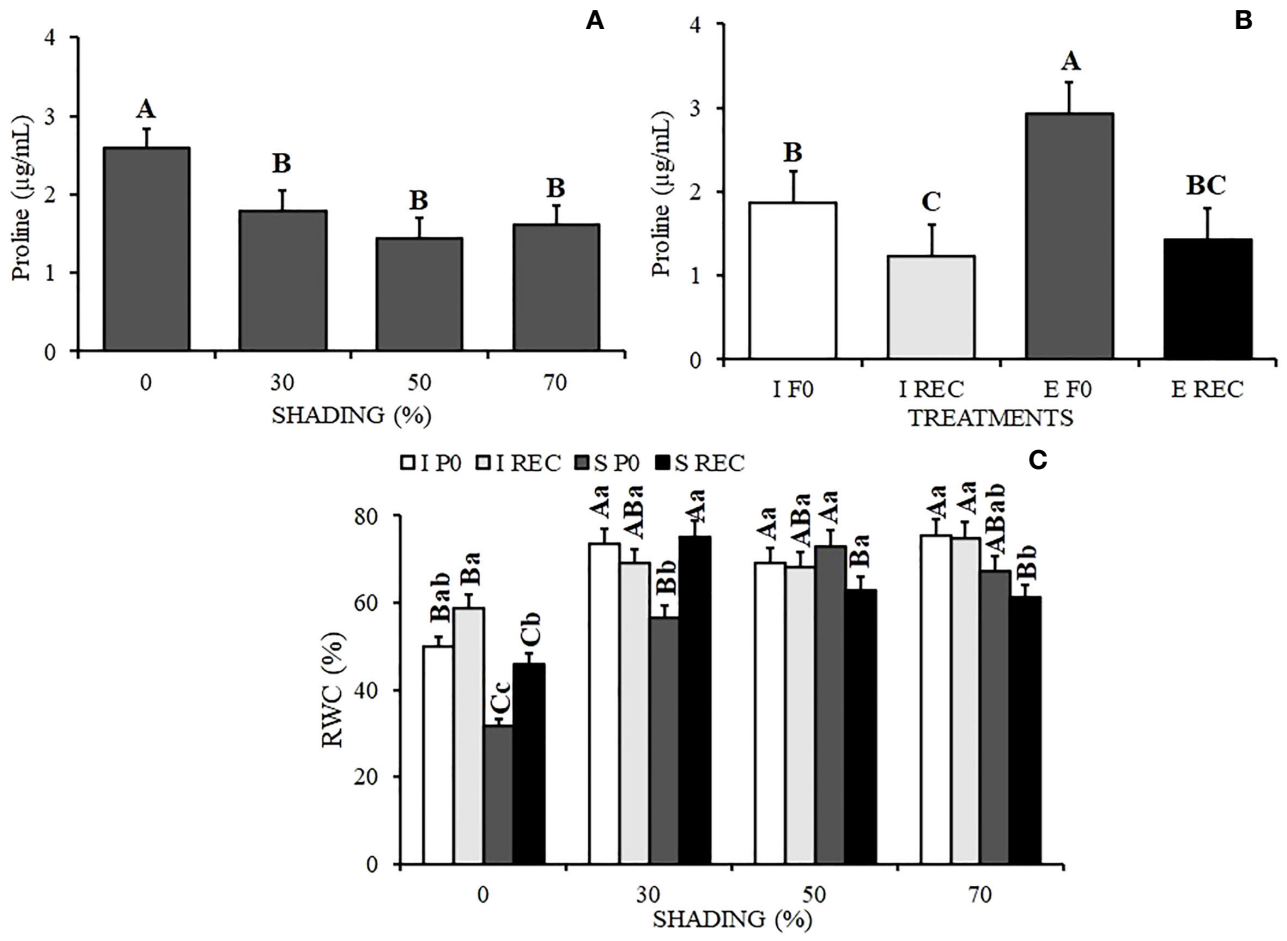
Mean Values ​​of Proline content **(A, B)** and Relative water content - RWC **(C)** in seedlings of *Hymenaea courbaril* L. under different water regimes+ evaluation periods: irrigated at zero photosynthesis (IP0), stressed at zero photosynthesis (SP0); irrigated in recovery (IREC), stressed in recovery (SREC) under the following different shading levels: 0 30, 50, and 70%. Means followed by different letters indicate significant differences by the Tukey`s test (p<0.05) (*n*= 3).

It is possible to observe an increase in proline in plants under 0% shading and also in the plants of the SP0 treatment (**Figures 5A, B**).

The relative water content in seedlings was lower under 0% shading and in SP0, followed by seedlings under 30% shading (**Figure 5C**). However, with the resumption of irrigation in the SREC, seedlings under 30% shading showed an increase in RWC, with values that did not vary from the control seedlings. This result differed from the plants under 0% shading, which showed an increase in RWC in relation to the previously stressed plants but presented statistically lower values than those of the plants of the control treatment.

**Figure 6:**
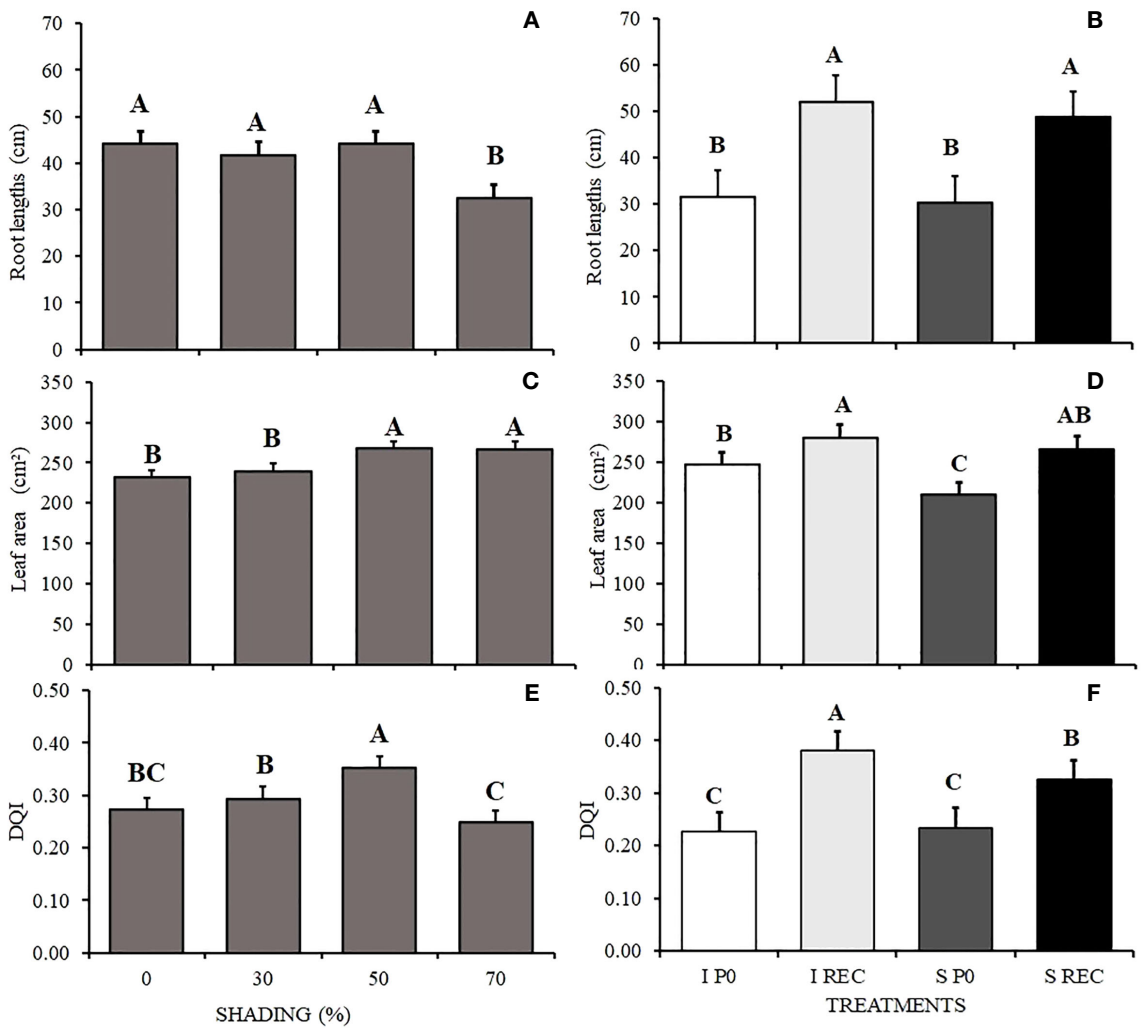
Mean values ​​of root lengths (CR) **(A, B)**, leaf area (AF) **(C, D)** and DQI **(E, F)** of *Hymenaea courbaril* L. seedlings under different water regimes+ periods of evaluation: irrigated at zero photosynthesis (IP0), stressed at zero photosynthesis (SP0); irrigated in recovery (IREC), stressed in recovery (SREC) under the following different shading levels: 0, 30, 50, and 70%. Means followed by different letters indicate significant differences by the Tukey`s test (p<0.05) (*n*= 3).

The smallest root length was observed in plants under 70% shading and in relation to the regimes and evaluation periods, it is possible to observe that in the SREC, regardless of the water regime, the plants presented a longer root length, with means that did not vary from each other (**Figures 6A, B**).

The leaf area was larger under 50 and 70% shading and smaller in plants under SF0, being to RWC, increasing in the SREC and not varying significantly between water regimes (**Figures 6C, D**).

The seedling quality (DQI) was higher in plants cultivated under 50% and lower in plants grown under 70% shading. For the water regimes and periods of evaluation, we observed lower a DQI in F0, regardless of the water regime. However, in the SREC, the highest quality was observed for plants of the IREC treatment (**Figures 6E, F**).

## Discussion

The harmful effect of water restriction on the activities of the photosynthetic apparatus was expected. However, the use of shading did not prevent the stressful effect of water deficit of reducing the *A*, *gs*, *A/E*, *A/C*_i_, F_v_/F_m_, and F_v_/F_0_ and increasing the *C*_i_, F_0_, and F_0_/F_m_ of plants of *H. courbaril*, nor was it essential for the recovery of these characteristics after re-irrigation in the REC, since there was recovery under all cultivation conditions. Thus, although shading did not prevent water deficit stress, it mitigated this stress and favored the recovery of the photosynthetic metabolism.

We emphasize that plants grown under shading would take more days to reach zero photosynthesis, taking into account that at the time of P0 observed only in plants under 0% shading, shaded plants showed higher values ​​of *A* (average values of 1.56, 2.27, and 1.61 µmol m^-2^ s^-1^) under 30, 50, and 70%, respectively. In addition, plants grown under 50% shading presented a recovery of the photosynthetic rate in three days, taking five days less than the plants under full sun, which only recovered after eight days of irrigation. Thus, 50% shading delayed the reduction of photosynthetic rate and anticipated the recovery of plants after re-irrigation.

Our results show that *H. courbaril* recovers photosynthetic metabolism quickly under favorable conditions. In a study carried out by different researchers with other tree and fruit species native to the Brazilian Cerrado, we observed that the recovery period of photosynthetic metabolism after water stress can vary from 12 to 13 days regardless of shading levels (0, 30, and 70%) for some species (Bartieres et al., 2020; Rosa et al., 2021) or 4 to 7 days under 30 to 40% shading in other (Bartieres et al., 2020; Rosa et al., 2021).

The lower stomatal conductance (*Gs)* and Rubisco carboxylation rate (*A/Ci*) and higher internal CO_2_ concentration of plants of *H. courbaril* under water deficit and 0% shading corroborate information in the literature in which these changes are related to variations in soil moisture due to irrigation, and in most species, reductions in *g*s associated with stomatal closure decrease water loss when exposed to low water availability and high insolation, while also reducing Rubisco activity (Reis et al., 2018; Bartieres et al., 2020; Freitas et al., 2020; Rosa et al., 2021; Wang et al., 2021). These authors suggested that the increase of *C*_i_ followed by reduction of *A* has been attributed to the lower rate of Rubisco carboxylation in plants under water and light stress.

We emphasize that there was no difference in the instantaneous water use efficiency (*A/E*) between shading during P0. However, in the REC, the *A/E* of plants under 0 and 30% shading increased. The maintenance of *A* associated with lower values of transpiration rate (*E*) are characteristics of plants that tolerate a lower availability of water in the soil, which reflects in a higher *A/E* (Ma et al., 2004).

The cultivation of plants of *H. courbaril* under 0% shading and water restriction represent a stressful condition for this species, which can be proven by the higher values of initial fluorescence (F0) and F_0_/F_m_ and lower values of F_v_/F_m_ and F_v_/F_0_, thus suggesting that both the biochemical and photochemical stages of photosynthesis were impaired by these environmental conditions.

The evaluations of parameters of chlorophyll *a* fluorescence have been used to investigate stressful conditions for seedlings. Although there are reference values for these parameters, it was observed in the literature that they can vary according to the species and its growth and metabolism potential. They observed that reference values for F_v_/F_m_ of 0.75-0.85 and for F_v_/F_0_ of 4-6 represent favorable conditions for cultivation, when the values of F_0_/F_m_ are below these values or above 0.14-0.20, it is suggested that the seedling is under a stressful condition (Bento et al., 2016; Reis et al., 2020; Rosa et al., 2021).

Thus, these parameters helped to identify the stressful condition caused by the irrigation suspension and full sun for the cultivation of plants of *H. courbaril*, which was reinforced by the behavior of the other results observed in these studies, such as the evaluations of carboxylation and stomatal conductance, reduction of protein content in leaves, increase in SOD activity in leaves and POX activity in leaves and roots, and increase in proline.

The increase in proline signals the stressful effect, although it did not mitigate the stress due to water deficit and direct exposure to the sun (0% shading), in the recovery after the resumption of irrigation, proline as an osmotic agent, favored cellular hydration and consequently the increase in stomatal conductance and photosynthetic rate of *H. courbaril* seedlings. Allied to this effect, we observed in the review by Szabados and Savouré (2009) that the synthesis of proline requires NADPH and generates NADP+ that can be used as an electron acceptor in photophosphorylation, maintaining the flow of electrons between the centers of photosynthetic excitation, stabilizing the equilibrium redox and damage to the photosynthetic apparatus and the production of energy necessary for the following stages of photosynthesis. Added to these effects, proline protects and activates antioxidant enzymes, such as SOD and POD, evaluated in this work, and other alternative detoxification pathways. In addition, the increase in energy provided by proline catabolism, in post-stress periods, allows the recovery of plant growth, as observed for the increase in root length, leaf area and DQI in *H. courbaril* plants after re-irrigation, in REC.

The growth characteristics evaluated in this study were not decisive to reinforce the water and light requirements of *H. courbaril*. Root length increased in REC, not varying depending on the water regimes between them, probably the time elapsed between the suspension of irrigation and P0 (10 days) was not enough to negatively affect the growth of the root system of this tree species, being a natural response of the plant. However, the reduction of leaf area in P0 may be a result of the harmful effect of water deficit. Both traits presented values statistically equal to the plants of the control treatment in the REC, indicating recovery potential.

We emphasize that the seedling quality indicates that *H. courbaril* presents a satisfactory growth under 50% shading, and that even after the stress caused by the water deficit, the plants resume growth. However, there was no total recovery in the evaluated period, since the DQI was significantly lower than that of the plants of the control treatment. Based on the other characteristics evaluated, we believe that the plants could also recover this quality if the evaluations were carried out in a slightly longer period.

It was evidenced that the shading better preserved hydration for leaves, which was confirmed by the greater RWC even in the time of greatest stress, as in P0. Shaded environments tend to be more humid, as the substrate surface presents less evaporation of water from the soil, which maintains the stomata open for longer, favoring greater CO_2_ assimilation and photosynthetic rate (Freitas et al., 2020). However, the more extreme shading certainly provided insufficient lighting for the growth needs of *H. courbaril*.

Bueno et al. (2021) observed that shading did not affect the height of plants of *H. courbaril*. However, the stem diameter was larger when grown under full sun (0% shading) and shading levels of 37 and 58%, the leaf area was larger and the biomass was higher under intermediate shading, and the highest quality (DQI) was observed under full sun and 37% shading, with significant reduction under 92% shading.

Our results corroborate the information from the literature, since the plants grown under very intense shading (70%) showed a reduced growth and quality, although maintaining high contents of chlorophylls a, b, and total chlorophyll, high growth of leaf area, and high water content in the leaves. The reduced growth was caused by the lower efficiency and carboxylation of Rubisco, which led to a lower photosynthetic rate and consequently to a lower production of photoassimilates to be invested in growth.

The increase in antioxidant enzymes, proline, and carotenoid contents and changes in chlorophyll *a* fluorescence caused by water deficit and by the sun proved that this species has protection mechanisms against these stressful conditions, which confers plasticity and survival potential in this stage of development.

*H. courbaril* is a climax species whose growth can be limited by low light levels (Oliveira et al., 2011). Thus, according to Pagliarini et al. (2017), the best plants are obtained under a shading screen of 30 and 50%. However, excess light is also harmful in this seedling stage. We emphasize that *H. courbaril* under full sun showed pioneer plant responses, such as an increase in carotenoids and alteration in photosystem II (PSII), although presenting low Rubisco activity and not presenting late-successional species responses, such as an irreversible decline in the maximum photochemical efficiency of PSII (F_v_/F_m_) or chlorophyll degradation.

Portela et al. (2019) observed that shade-tolerant tropical species can survive in the sun or under intermediate shade and that, regardless of successional class, climax or pioneer species grow better under intermediate shade, although tolerating direct light. Under direct light, climax plants present high survival but low growth, which is attributed to their high plasticity.

In view of the above and what was observed in our study, corroborating what was observed in the literature, we also found that this species has great potential to be used in projects for the recovery and enrichment of degraded areas subject to periods of water deficiency, since it has the ability to recover its photosynthetic metabolism and resume its growth, although shade cultivation minimizes the stressful effect of water deficit and favors the recovery of seedlings.

## Conclusion

Intermediate shading of 30 and 50% with 700 and 500 μmol m^-2^ s^-1^ PPFD mitigates the water deficit and accelerates the recovery of *H. courbaril*.

Water deficit associated with cultivation without shading (0%) with 1,300 μmol m^-2^ s^-1^ PPFD should not be adopted in the cultivation or transplantation of plants of *H. courbaril*.

When shaded and re-irrigated, the previously stressed plants can recover their photosynthetic metabolism without changes and/or permanent damage to the efficiency of photosystem II, presenting the resumption of growth and seedling quality, which proves the hypothesis of this study.

## Data availability statement

The original contributions presented in the study are included in the article/supplementary material. Further inquiries can be directed to the corresponding author.

## Author contributions

LR, AF VL collected data, LR, DD, CS conducted statistical analysis, SS, DD, CS supervised the experimental work and all authors wrote the article. All authors contributed to the article and approved the submitted version.
